# Atypical seasonal variability of the Kuroshio Current affected by intraseasonal signals at its origin based on direct mooring observations

**DOI:** 10.1038/s41598-022-17469-5

**Published:** 2022-07-30

**Authors:** Fujun Wang, Linlin Zhang, Junqiao Feng, Dunxin Hu

**Affiliations:** 1 Key Laboratory of Ocean Circulation and Waves, Institute of Oceanology, Chinese Academy of Sciences, Qingdao, China; 2 Center for Ocean Mega-Science, Chinese Academy of Sciences, Qingdao, China; 3 Laboratory for Ocean and Climate Dynamics, Qingdao National Laboratory for Marine Science and Technology, Qingdao, China

**Keywords:** Hydrology, Ocean sciences

## Abstract

The spatial distribution and temporal variability of the Kuroshio Current (KC) was investigated with three moorings deployed at 122.7° E, 123° E, and 123.3° E along 18° N from January 2018 to the spring of 2020. It is shown that the core of the KC is located to the west of 122.7° E along 18° N. With the increase in longitude, the KC extended its vertical scale and attenuated its intensity gradually. The satellite data indicated that the KC was strongest in winter and spring, while it was weakest in autumn along 18° N. However, the seasonal cycle of the KC from mooring observations was atypical compared with that from the satellite data. The seasonal variation of the KC was not obvious in 2018, and a summer peak of KC occurred in 2019. The atypical seasonal variability of the KC was attributed to the strong intraseasonal signals generated by eddy activity. Eddies propagated from east and were enhanced to the west of 140° E, leading to the westward intensified intraseasonal signals. In addition, the intraseasonal signals varied interannually, that is why the variation of the KC in 2018 was quite different with that in 2019.

To the east of Mindanao Island, the westward flow of the North Equatorial Current (NEC) bifurcates into the southward Mindanao Current (MC)^1–4^ and the northward Kuroshio Current (KC)^5–8^. Beneath the KC, the Luzon Undercurrent (LUC) flows southward intermittently^9–11^. At its origin, the KC flows almost parallel to the coastline of the Philippines. To the north of Luzon Island, part of the KC intrudes into the South China Sea (SCS) via the Luzon Strait^12–15^, while the rest flows northeast along the east coast of Taiwan^2^. At approximately 24.5° N, the KC enters the East China Sea (ECS) via the East Taiwan Channel, and then moves toward the southeast coast of Japan. After separating from the Japanese coast, the KC enters the Kuroshio Extension (KE), which is characterized by energetic eddies and meanders^16–19^. The KC plays an important role in the exchange of heat, salt, and water between tropical and extratropical oceans along its path, and influences both local and global climate^20,21^.

Along the entire route of the KC, its strength and vertical structure vary significantly with respect to time and space. Intraseasonal, seasonal, interannual, and decadal variations of the Kuroshio path have been observed in previous studies (e.g., Refs.^18,22–28^. Using the mean monthly geostrophic current from satellite data along 18.75° N, Lien et al.^29^ pointed out that the Kuroshio transport is stronger (weaker) in winter and spring (summer and autumn). Nakamura^30^ reviewed the temporal variability of the KC and showed that the seasonal KC is similar in the region east of Luzon Island, east Taiwan, and in the ECS, but the interannual variability of the KC is different along its path. Based on high-resolution hybrid-coordinate ocean model reanalysis data for 1992–2016, Chen et al.^31^ demonstrated that large-scale wind forcing in the western tropical Pacific Ocean, rather than the local wind forcing, plays a dominant role in the decadal variation of the Kuroshio intrusion into the SCS.

In the poleward route of the KC, intraseasonal variability always depends significantly on eddy activity. Generally, mesoscale eddies are generated in the interior ocean and propagate westward to the western boundary^32,33^. The mesoscale eddies to the east of Luzon and Taiwan are attributed to the baroclinic instability between the North Pacific Subtropical Countercurrent (STCC) and the NEC system^34–36^. It has been shown that anticyclonic (cyclonic) eddies strengthen (weaken) the transport of the KC to the east of Luzon and Taiwan^37^.

Although there are some mooring measurements for the KC to the east of Taiwan (e.g., Refs.^38–41^), long-term direct observations from mooring arrays in the source region of the KC are relatively sparse (e.g., Refs.^10,29,42^. Three moorings at 122.7° E, 123° E, and 123.3° E along 18° N are utilized to discuss the mean structure, seasonal variability, intraseasonal variability of the KC, and then to clarify the influence of intraseasonal variability on the seasonal cycle of the KC in the present study.

## Data and methods

**Figure 1:**
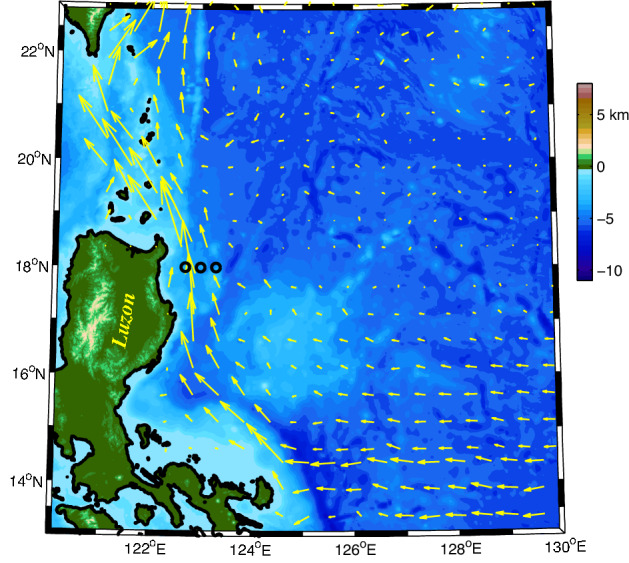
Bottom topography in the origin region of the KC. Black circles denote the moorings deployed at 122.7° E, 123° E, and 123.3° E along 18° N. Color shows the bathymetry of the western Pacific Ocean, which is downloaded from https://download.gebco.net/. The arrows represent the mean geostrophic currents (January 1993 to December 2019) from satellite altimetry (http://www.marine.copernicus.eu). Figures are plotted using MATLAB R2012b (http://www.mathworks.com/).

In January 2018, three moorings were deployed at 122.7° E, 123° E, and 123.3° E along 18° N to the east of the Philippines with the R/V *Science* (Fig. 1). Each mooring had an upward looking acoustic doppler current profiler (ADCP) and a downward looking ADCP. The moorings were then retrieved successfully in May 2020 with the R/V *Science 3* during a research voyage of the Institute of Oceanology, Chinese Academy of Sciences (IOCAS). The three moorings were designed to clarify the spatial distribution and temporal variability of the KC/LUC as well as the role of eddy activity in this region where the water depths are 3124 m, 3383 m, and 3437 m at 122.7° E, 123° E, and 123.3° E along 18° N, respectively. Unfortunately, one of the downward-looking ADCPs at longitude 123° E was broken. Table 1 provides information about the three moorings.

**Table 1:** Positions, total days, and corresponding periods for the ADCPs during mooring observations.

Position	Days	Period
122.7° E/18° N	828	01/25/2018–05/01/2020
123° E/18° N	766	01/25/2018–02/29/2020
123.3° E/18° N	790	01/25/2018–03/24/2020

In detail, upward and downward looking 75 kHz ADCPs were fixed on the main float of each mooring. Each ADCP was set as 60 bins with 8 m per bin; thus, its measurement range was 480 m. As the main float was designed at 450 m, the velocities in the upper 900 m could be well monitored by two ADCPs. We note that the strength of the KC led to an incline of the mooring system, suggesting that the real measurement range was < 900 m. The mooring data with 8 m intervals were interpolated into 1 m resolution. For data quality control, the velocity magnitude was limited to 3 m/s, the percent good beam in ADCPs with a quality of > 80 were accepted, and the data with > 18° of pitch and roll were deleted. We note that the velocities in the upper 50 m of the water column were deleted owing to poor quality data that resulted from the strong echo of beam emission. The velocity resolution of ADCP is 1 mm/s, and the velocity accuracy is ± 1% ± 5 mm/s. As the ADCP data were collected on an hourly basis, it was convenient to convert the mooring data to a daily time series to remove tidal signals.

In addition, a global sea surface height (SSH) dataset from the Copernicus Marine and Environment Monitoring Service (CMEMS) was utilized in this study. This dataset merges SSH observations from all satellite altimeter missions, such as the Ocean Topography Experiment Topex/Poseidon, European Remote Sensing Satellite-1 (ERS-1), ERS-2, Geosat Follow-On, Jason-1, and Jason-2. The daily SSH and corresponding geostrophic current data with a resolution of 0.25° × 0.25° from January 1, 1993, to December 31, 2019, were derived from http://www.marine.copernicus.eu.

## Results

### Mean structure of the KC

**Figure 2:**
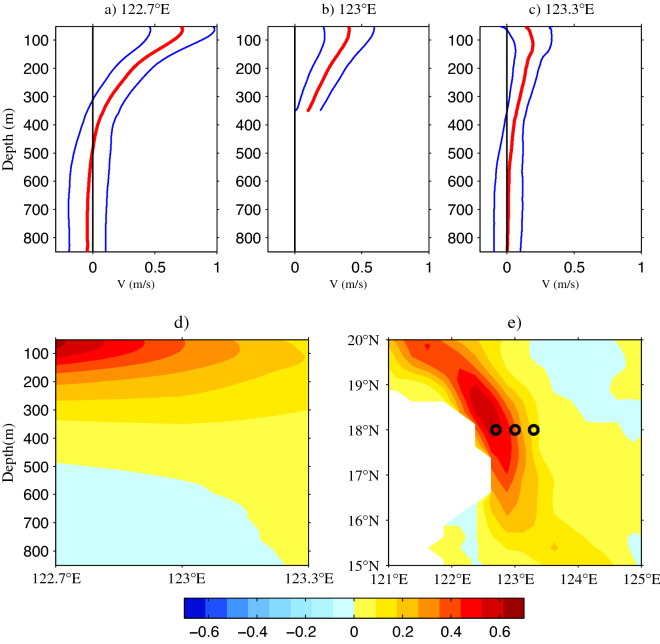
Mean meridional velocities (red lines, m/s) and corresponding standard deviations (blue lines) at (**a**) 122.7° E, (**b**) 123° E, and (**c**) 123.3° E along 18° N. The black lines represent zero velocity. (**d**) Longitude–depth meridional velocities of three moorings at 122.7° E, 123° E, and 123.3° E along 18° N. (**e**) Mean meridional velocities calculated from satellite altimeter data (1993–2019) in the origin region of the KC (units are m/s). Figures are plotted using MATLAB R2012b (http://www.mathworks.com/).

Around 18° N, the direction of the KC is almost parallel to the coastline of Luzon Island (Fig. 1); therefore, the meridional velocities collected by the ADCPs are suitable for representing the KC. In this study, the northward and eastward velocities were defined as positive. The mean meridional velocities and corresponding standard deviations at 122.7° E, 123° E, and 123.3° E along 18° N are shown in Fig. 2a–c. At the sea surface, the mean meridional velocity attenuated with an increase in longitude, reducing from 70.7 cm/s at 122.7° E via 40.3 cm/s at 123° E to 14.7 cm/s at 123.3° E, indicating that the core of the KC was located to the west of 122.7° E. The maximum mean velocity at 122.7° E, 123° E, and 123.3° E was 72.4 cm/s at a depth of 63 m, 40.6 cm/s at a depth of 62 m, and 19.5 cm/s at a depth of 115 m, respectively. The zero meridional velocity occurred at 488 m and > 800 m at 122.7° E and 123.3° E along 18° N, respectively. The maximum standard deviation was 25.8 cm/s at a depth of 63 m, 19.0 cm/s at a depth of 51 m, and 19.9 cm/s at a depth of 51 m, respectively, suggesting that the variability of the KC was mainly focused in the upper layer. We note that the standard deviation decreased with depth until ~ 400 m before increasing, which could be attributed to the fact that the LUC is under ~ 400 m.

The longitude-depth profile interpolated by the three moorings is displayed in Fig. 2d. Notice that it is interpolated by three moorings in the upper 350 m and two moorings (122.7° E and 123.3° E) below 350 m, as the downward looking ADCP at 123° E is broken. The strength of the KC decreased with longitude and depth, suggesting that the core of the KC was located to the west of 122.7° E at 18° N. With the increase in longitude, although the strength of the KC decreased, its vertical scale extended. In order to depict the spatial distribution of the KC, the mean surface meridional velocity for 1993–2019 was determined using satellite data, as shown in Fig. 2e. The KC core was limited to 1° east of the Philippines and flowed northwestward along the coastline. The core was located at 122.625° E/18.125° N, which was very close to the mooring station at 122.7° E/18° N. Lien et al.^32^ used ADCP moorings and pressure inverted echo sounders (PIES), and found that the axis of the KC was located at ~ 122.4° E along 18.75° N.

### Seasonal and intraseasonal variability of the KC

**Figure 3:**
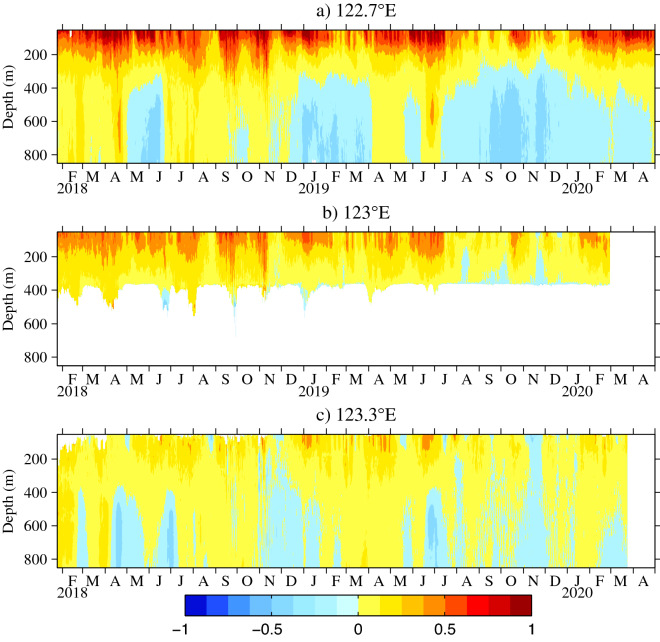
Daily meridional velocities from ADCPs at (**a**) 122.7° E (January 2018 to May 2020), (**b**) 123° E (January 2018 to February 2020), and (**c**) 123.3° E (January 2018 to March 2020) along 18° N (units are m/s). Figures are plotted using MATLAB R2012b (http://www.mathworks.com/).

The daily meridional velocities from the ADCPs at 122.7° E, 123° E, and 123.3° E along 18° N are presented in Fig. 3. The KC often exceeded 1 m/s at all three stations during the study period. Generally, the magnitude of the KC decreased from 122.7° E to 123.3° E, which is consistent with the results in Fig. 2a–c. During the observational time series, the KC was always present in the upper 400 m. In 2019, the KC was strongest during the winter, spring and summer, and weakest during the autumn at all three stations. However, the seasonal cycle was not significant at all stations in 2018, suggesting that the intraseasonal signals play an important role during 2018 and have the interannual variability. At 122.7° E and 123° E, the magnitude of the KC was stronger in 2018 than that in 2019, but this was reversed at 123.3° E. It is obvious that the intraseasonal signals were enhanced from 123.3° E westward to 122.7° E along 18° N. Beneath the permanent northward-flowing KC, the southward-flowing LUC existed intermittently. At 122.7° E in 2018, The LUC existed only in May and June. In contrast, the LUC disappeared only in August, May, late June and early July in 2019, and it was weakest in summer. Unlike the KC, the magnitude of the LUC in 2019 was stronger than that in 2018. At 123.3° E, the intraseasonal signals of the LUC were obvious in 2018.

**Figure 4:**
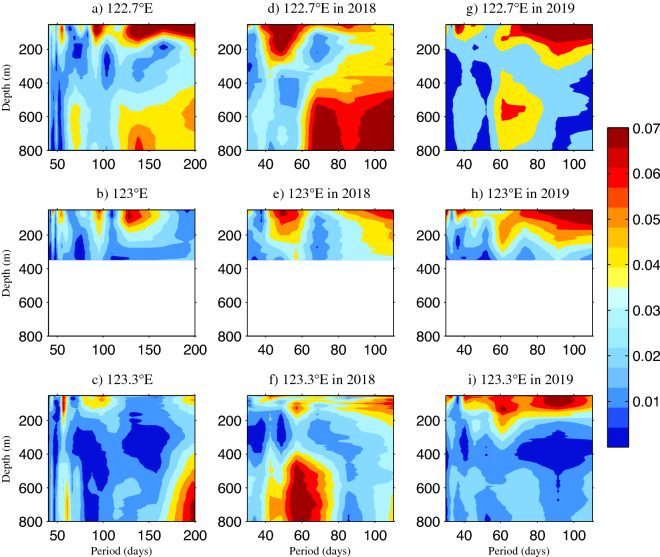
Power spectral density (PSD) of the meridional velocities during the whole observational period at (**a**) 122.7° E, (**b**) 123° E, and (**c**) 123.3° E, and during 2018 at (**d**) 122.7° E, (**e**) 123° E, and (**f**) 123.3° E, and during 2019 at (**g**) 122.7° E, (**h**) 123° E, and (**i**) 123.3° E along 18° N. Units are m^2^/(s^2^ cycles per day [cpd]). Figures are plotted using MATLAB R2012b (http://www.mathworks.com/).

The 2 years mooring data are convenient to focus on the intraseasonal variability under a power spectrum density (PSD) analysis. It is shown in Fig. 4a–c that significant periods of 50–60 days and 80–100 days were common in the upper layers of all three mooring stations. To explicitly evaluate the intraseasonal signals in different years, a PSD analysis was applied to the three stations in 2018 and 2019, respectively (Fig. 4d–i). The period of 50–60 days was much stronger in the upper 350 m during 2018 in comparison to 2019 at three mooring stations. This suggests that the intraseasonal signals varied interannually, which is consistent with the above analysis. We note that the magnitude of the period of 50–60 days attenuated from 122.7° E to 123.3° E, which indicates that the intraseasonal signals are stronger towards the west. It is noted that the inraseasonal signals were significant below 400 m at 123.3° E in 2018.

**Figure 5:**
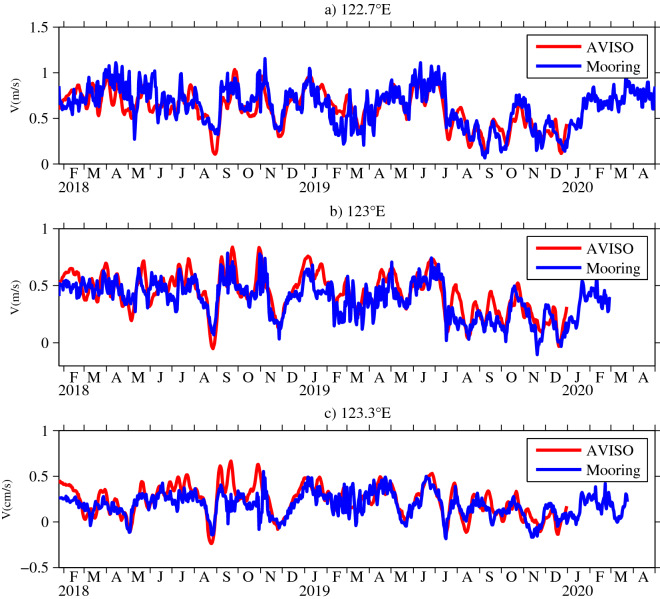
Observational meridional velocities (averaged over the upper 150 m) and the geostrophic currents from satellite data at (**a**) 122.7° E, (**b**) 123° E, and (**c**) 123.3° E along 18° N (units are m/s). The geostrophic currents are calculated from satellite altimetry (http://www.marine.copernicus.eu). Figures are plotted using MATLAB R2012b (http://www.mathworks.com/).

The temporal and spatial resolutions of the mooring data were insufficient and limited to 2 years and three stations. Our calculation shows that the geostrophic velocities were very consistent with the mooring observations averaged over the upper 150 m, both with respect to amplitude and phase (Fig. 5). These findings suggest that satellite altimeter data are convenient for investigating the seasonal and intraseasonal variability of the KC. Moreover, these findings also indicate that the direct observations could be considered as geostrophic currents in the origin region of the KC. This is consistent with the results of Lien et al.^29^, who found that the geostrophic theory could be used to describe the KC (except in the upper 100 m of the KC’s western flank).

**Figure 6:**
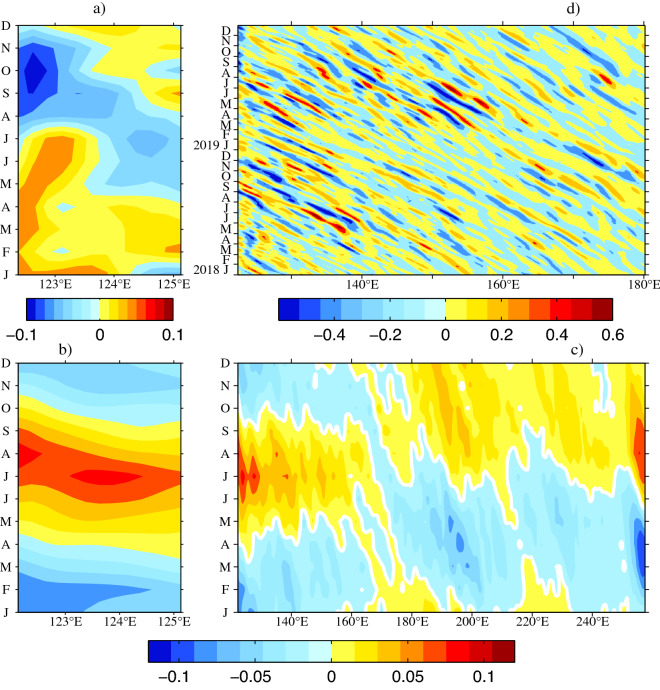
(**a**) Mean monthly meridional velocity anomalies (January 1993 to December 2019), and mean monthly SSHa (January 1993 to December 2019) from satellite altimeter data along 18° N (**b**) in the origin region of the KC and (**c**) across the North Pacific Ocean (units are m), and (**d**) the meridional velocity anomalies from January 2018 to December 2019 calculated from satellite altimeter data along 18° N (units are m/s). The geostrophic current anomalies are calculated from satellite altimetry (http://www.marine.copernicus.eu). Figures are plotted using MATLAB R2012b (http://www.mathworks.com/).

In Fig. 6a, the mean monthly meridional velocities based on satellite data indicate that the KC was strongest in winter and spring, and weakest in autumn during the period from 1993 to 2019. Notice that the seasonal variation of the KC is different along Luzon Strait. For example, Zhang et al.^27^ indicates a July maximum of the KC in the northern flank of Luzon Strait. The mean monthly sea surface height anomaly (SSHa) averaged from 1993 to 2019 is shown in Fig. 6b. Two adjacent zonal points (${x}_{1}$ and ${x}_{2}$) in the origin region of the KC along 18° N were chosen to discuss the phase difference of the zonal SSHa. The maximum SSHa was in August at 121° E, while it occurred in July at ~ 123.7° E, so we set ${x}_{1}$ = 121° E and ${x}_{2}$ = 123.7° E. As the propagation speed of Rossby waves near the western boundary along 18° N is approximately 0.11 m/s^43^, $\Delta t=\frac{{x}_{2}-{x}_{1}}{{c}_{r}}\approx$ 1 month. This result is consistent with the fact that the maximum SSHa at 123.7° E required nearly 1 month to arrive at 121° E, as shown in Fig. 6b, suggesting that Rossby waves process is one of the possible dynamical processes in the seasonal variability of the KC. At all mooring stations, the zonal gradient of the SSHa was always positive from December to July (Fig. 6b), generating a northward current anomaly that increased the KC. In contrast, the zonal gradient of the SSHa was negative from August to November, corresponding to a southward current anomaly that decreased the KC. In addition, the mean monthly SSHa across the Pacific Ocean along 18° N is depicted in Fig. 6c. It is worth emphasizing that the spatial distribution of the SSHa along 18° N differed considerably to the east and west of the dateline. To the east of the dateline (180° E), the positive SSHa occurred during July–December, and the negative SSHa existed during January–June. However, to the west of the dateline, the positive SSHa occurred during May–October, and the negative existed during November–April. This difference may have been strongly influenced by the longitude-dependent spatial distribution of wind forcing in the North Pacific Ocean. Zhang et al.^28^ showed that the local wind plays an important role in seasonal upper-layer velocity variations of the KC. Thus, the seasonal KC is the result of the local wind, Rossby waves, surface heat flux^44,45^ and so on.

However, it is obvious that the seasonal variation derived from satellite data in Fig. 6a is different from mooring observations especially in 2018 in Fig. 3. The meridional velocity anomalies from January 2018 to December 2019 calculated from satellite altimeter data along 18° N are shown in Fig. 6d. It is noted that the magnitude of seasonal variability in Fig. 6a is smaller than the intraseasonal signals in Fig. 6d, suggesting that the intraseasonal variability is more significant than the seasonal variability during mooring observations. In fact, the origin of the KC is a rich eddy region where the intraseasonal variability of the KC is strongly influenced by eddy activity^35,42^. Obviously, that eddies propagated westward and were enhanced to the west of 140° E along 18° N in Fig. 6d, well corresponding to the westward intensified intraseasonal signals. In addition, the behavior of intraseasaonal signals in 2018 were different from that in 2019, indicating that the intraseasonal signals varied interannually. For example, the positive maximum of the SSHa occurred at the mooring stations in September and October 2018, resulting in a strong northward flow anomaly. In 2019, strong anticyclonic eddies from the east arrived at the mooring stations in June and July, thus generating a summer peak of the KC.

## Conclusion and discussion

The seasonal and intraseasonal variabilities of the KC in its origin region were investigated using satellite altimeter data and direct mooring observations at 122.7° E, 123° E, and 123.3° E along 18° N. The KC extended its vertical scale and attenuated its intensity gradually with increased longitude. Beneath the permanent northward KC, the intermittent southward LUC led to an increase in the standard deviation of the current velocity below a depth of 400 m. Within the observational time series, the KC often exceeded 1 m/s.

The satellite data indicated that the KC was strongest in winter and spring, and weakest in autumn along 18° N. The consistency between the mooring data and the meridional velocities calculated from satellite altimeter data indicates that the KC at the three mooring stations maintained a geostrophic balance during the study period. It is shown that the SSHa gradually propagates toward the west; for example, there is a ~ 1 month interval between 121° E and 123.7° E, thus generating a zonal gradient of the SSHa. This situation leads to a negative zonal gradient from August to November, and a positive gradient from December to July, thereby producing a complete seasonal cycle of the KC.

The mooring observations showed that the seasonal variability of KC was not obvious and the intraseasonal signals played an important role in 2018, which is confirmed from the PSD that the intraseasonal signals were stronger in the upper 350 m in 2018 than in 2019. The origin of the KC is a rich eddy region where the intraseasonal variability of the KC is strongly influenced by eddy activity^35,42^. These eddies propagated from the east and were enhanced to the west of 140° E.

**Figure 7:**
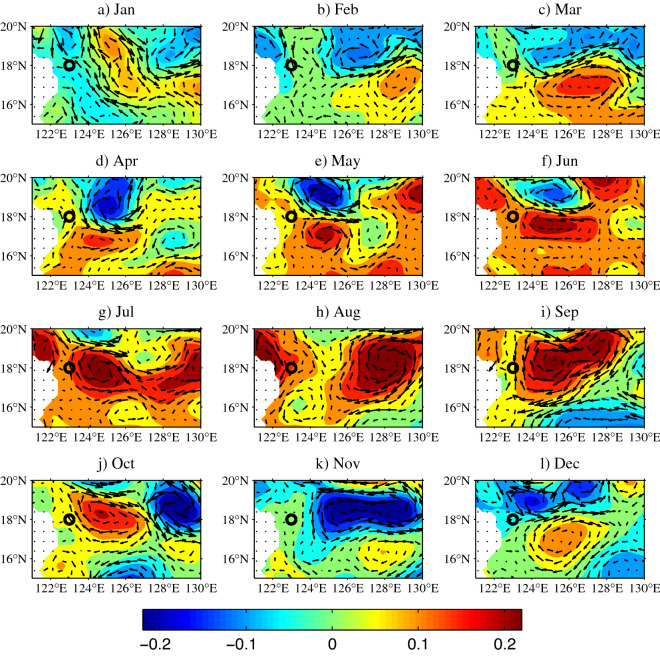
SSHa (m) from satellite altimeter data in the origin region of the KC in (**a**) January, (**b**) February, (**c**) March, (**d**) April, (**e**) May, (**f**) June, (**g**) July, (**h**) August, (**i**) September, (**j**) October, (**k**) November, and (**l**) December 2018. Black circles denote the mooring deployed at 123° E, 18° N. The geostrophic current anomalies are calculated from satellite altimetry (http://www.marine.copernicus.eu). Figures are plotted using MATLAB R2012b (http://www.mathworks.com/).

To depict the spatial structure of the SSHa variability more clearly, the SSHa in 2018 in the region of 121°–130° E/15°–20° N is shown in Fig. 7. The arrows represent the corresponding geostrophic current anomalies. It reveals that the cyclonic and anticyclonic eddies simultaneously occupied the region to the east of the mooring stations from March to June, and in December 2018. The mooring stations were located in the western flank of these two eddies. The interaction of these two eddies meant that the northward and southward current anomalies alternately occupied the mooring stations, suggesting a relatively higher frequency compared with that without eddies in the origin region of the KC. Gordon et al.^42^ pointed out that there are two energetic dipoles bracketing a northward flowing stream into the KC. The cyclonic dipole occupies the southern tier of Lamon Bay, while the center of the anticyclonic dipole may lie at 17° N/125° E. In September and October in Fig. 7, an anticyclonic circulation occurred in the area to the east of our mooring stations, which almost coincided with the anticyclonic dipole mentioned above, and resulted in a northward current anomaly that increased the KC. Obviously, eddies propagated westward and reduced when they arrived at the western boundary. In this process, intraseasonal variability of the KC is controlled by the propagation and reduction of eddies. Therefore, intraseasonal signals are important and cannot be ignored in the seasonal cycle of eddy-rich years in the origin region of the KC.
